# Correcting Principal Component Maps for Effects of Spatial Autocorrelation in Population Genetic Data

**DOI:** 10.3389/fgene.2012.00254

**Published:** 2012-11-20

**Authors:** Eric Frichot, Sean Schoville, Guillaume Bouchard, Olivier François

**Affiliations:** ^1^Université Joseph Fourier Grenoble, Centre National de la RechercheGrenoble, France; ^2^Xerox Research Center EuropeMeylan, France

**Keywords:** principal component analysis, isolation-by-distance, spatial autocorrelation, spatial factor analysis

## Abstract

In many species, spatial genetic variation displays patterns of “isolation-by-distance.” Characterized by locally correlated allele frequencies, these patterns are known to create periodic shapes in geographic maps of principal components which confound signatures of specific migration events and influence interpretations of principal component analyses (PCA). In this study, we introduced models combining probabilistic PCA and kriging models to infer population genetic structure from genetic data while correcting for effects generated by spatial autocorrelation. The corresponding algorithms are based on singular value decomposition and low rank approximation of the genotypic data. As their complexity is close to that of PCA, these algorithms scale with the dimensions of the data. To illustrate the utility of these new models, we simulated isolation-by-distance patterns and broad-scale geographic variation using spatial coalescent models. Our methods remove the horseshoe patterns usually observed in PC maps and simplify interpretations of spatial genetic variation. We demonstrate our approach by analyzing single nucleotide polymorphism data from the Human Genome Diversity Panel, and provide comparisons with other recently introduced methods.

## Introduction

The concept of “isolation-by-distance” (IBD) was introduced by S. Wright to describe the accumulation of local genetic differences under spatially restricted dispersal (Wright, 1943). In species that are continuously distributed in geographic space and disperse over short distances, the theory predicts that genetic differentiation will increase with geographic distance (Malécot, 1948; Kimura and Weiss, 1964). IBD can be described by spatial autocorrelation, a measure of the degree of dependency among observations in a geographic space. Although studying IBD patterns could lead to useful estimates of gene dispersal (Rousset, 1997), spatial autocorrelation derived from IBD often presents a problem for population genetic analyses. More specifically, the presence of spatial autocorrelation patterns can increase the rate of false positive tests for hierarchical population structure or for the detection of loci under selection (Meirmans, 2012).

Recently, it has been acknowledged that distortions caused by spatial autocorrelation could also bias interpretations of population genetic structure as inferred from principal component analysis (PCA) or from Bayesian clustering methods (Novembre and Stephens, 2008; François et al., 2010). PCA is a method that searches for axes, called principal components, along which projected individuals show the highest variance. As a result, the first PCs are often used to explore the structure of variation in the sample. Characterized by locally correlated allele frequencies, IBD patterns create periodic shapes in PC maps that can confound signatures of migration events and influence interpretations of principal component analyses (Novembre and Stephens, 2008). In scenarios where covariance decays exponentially with geographic distance, PC plots are indeed expected to exhibit horseshoe effects, an artifact in which the second axis is curved relative to the first axis. These effects lead to counterintuitive representations of the data (Legendre and Gallagher, 2001; Diaconis et al., 2008).

Several methods have been proposed to correct for the effects of spatial autocorrelation in exploratory data analyses. In particular, those methods include spatial Principal Component Analysis (sPCA, Borcard and Legendre, 2002; Borcard et al., 2004; Dray et al., 2006; Jombart et al., 2008), and sparse factor analysis (SFA, Engelhardt and Stephens, 2010). Generally the methods share the objective of separating local and regional geographic scales in the data. In this study, we introduce a novel approach, based on latent factors models, that addresses the separation of geographic scales more directly than the two previous methods. The new method, spatial factor analysis (spFA), combines probabilistic PCA (Tipping and Bishop, 1999) and kriging models (Cressie, 1993) to infer population genetic structure from genetic data while correcting for errors introduced by spatial autocorrelation. While many approaches have been argued to improve interpretations of the data, their outputs have not yet been compared to each other on the basis of spatial simulations. To compare methods, we generated patterns of IBD and broad-scale geographic variation using computer simulations of spatial coalescent models. We compared the outcomes of methods under population genetic models of isolation-by-distance, and we argued that the methods provided insights on distinct aspects of the data. We report that the new spFA method was able to remove the horseshoe effect observed in spatially structured data, whereas this was not the case in PCA, sPCA, and SFA analyses. We discuss the significance of this result in an assessment of single nucleotide polymorphism data from worldwide samples of the Human Genome Diversity Panel.

## Materials and Methods

We considered single nucleotide polymorphism (SNP) data for *n* individuals genotyped at *L* loci. For these data, the genotypic matrix entries, (*G_il_*), record the number of derived alleles at locus *l* for individual *i*. For autosomal data, *G_il_* is thus equal to 0, 1, or 2, and corresponds to the genotype at locus *l*. The data were centered by subtracting the mean value of each column of *G* and scaled by dividing by the standard deviation value of each column of *G*. In addition to the genotypic data, we assumed that geographical coordinates, (*X_i_*), were recorded for each individual.

We evaluated the effects of IBD patterns on inference of population genetic structure using 4 statistical methods: Principal Component Analysis (PCA, Jolliffe, 1986; Patterson et al., 2006), spatial PCA (sPCA, Jombart et al., 2008), Sparse Factor Analysis (SFA, Engelhardt and Stephens, 2010), and a new method called *spatial Factor Analysis* (spFA).

### Principal component analysis

PCA is a popular method that searches for a set of *K* orthogonal axes (the principal components), each of which is a linear combination of the original axes, such that projections of the original data display maximal variance onto the new axes (McVean, 2009). We computed the score matrix, *U* of dimension *n* × *K*, and the loading matrix, *V* of dimensions *K* × *L*, using the rank *K* singular value decomposition method implemented in the R function prcomp and in the computer program *SmartPCA* (Patterson et al., 2006).

### Moran eigenvectors and spatial PCA

Moran eigenvectors maps were proposed as an alternative to trend surface analysis for incorporating spatial variation in population genetics models (Dray et al., 2006; Jombart et al., 2008). In Moran eigenvectors maps, there are positive and negative eigenvalues. Eigenvectors associated with positive eigenvalues have positive autocorrelation, and they describe global structures. Eigenvectors associated with negative eigenvalues describe local structures. Implemented in an algorithm called spatial PCA (sPCA), Moran’s eigenvector maps (MEM) maximize Moran’s spatial autocorrelation index, defined as follows

$$I\left(G\right)=\frac{\sum_{i,j}w_{ij}\left(g_{i}-ḡ\right)\left(g_{j}-ḡ\right)\;}{\sum_{i,j}w_{ij}\sum_{i}{\left(g_{i}-ḡ\right)}^{2}}$$

with respect to a spatial weighting matrix, *W*, deduced from geographical distances and where $g_{i}$ is the *i*th column of *G* (Dray et al., 2006). We implemented MEMs and sPCA using the R package adegenet using a Delaunay weighting matrix (Jombart et al., 2008).

### Spatial factor analysis

We introduce a new spatial factor analysis model (spFA) which incorporates spatial information in factor analysis in an explicit way. In spFA, inference is performed in a matrix factorization model similar to probabilistic PCA (Tipping and Bishop, 1999).

(1) $$G_{i\ell }=U_{i}^{T}V_{\ell }+\epsilon_{i\ell },$$

where *ε_iℓ_* are statistically dependent Gaussian variables with mean zero and with covariance matrix Σ*_θ_*. Similarly to Kriging approaches (Cressie, 1993), a radial basis covariance matrix was chosen to model spatial autocorrelation patterns generated by IBD (see also Durand et al., 2009). The covariance matrix Σ*_θ_* was defined as follows. For all pairs of individuals, *i* and *j*, we have

(2) $$\sum_{\theta }\left(i,j\right)=\exp\left(-d\left(X_{i},X_{j}\right)/\theta \right),\;\theta >0,$$

where *d*(*X_i_*, *X_j_*) represents the squared Euclidean or great-circle distance between sites with coordinate *X_i_* and with coordinate *X_j_*. To avoid collinearity issues, we assumed that the individual geographical coordinates were distinct from each other (ties were broken by adding small perturbations to the original spatial coordinates). The parameter *θ* is a scale parameter measured in units of average pairwise distance between geographic sites, $\bar{d}$ In practice, spFA requires that an array of *θ* values (scale parameter) are explored, so *θ* was varied in the range $\left(0,\;10\bar{d}\right).$

**Figure 1:**
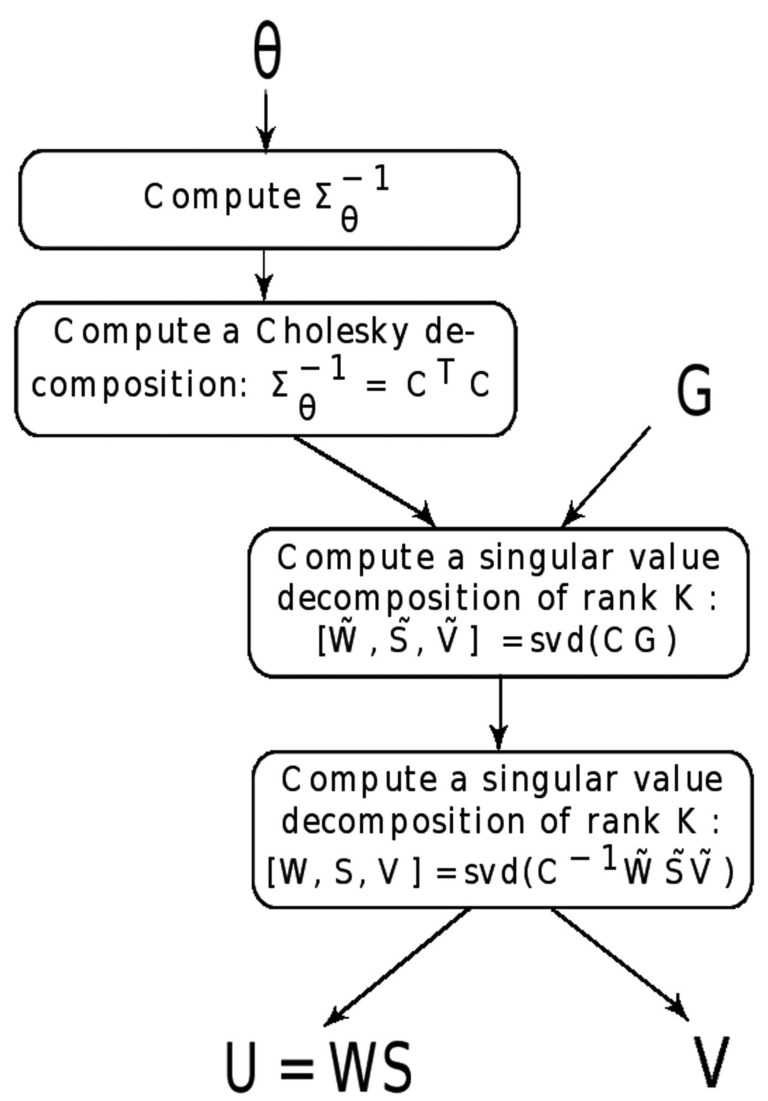
**Algorithm for spFA**. For a genotypic matrix *G* with individual geographic coordinates (*X_i_*), and for scale parameter *θ* > 0, the spFA steps summarize as follows.

To solve the spFA model, we used a Cholesky decomposition, $C^{T}C=\Sigma_{\theta }^{-1},$ and we established an equivalence with the following matrix factorization model

(3) $${\tilde{G}}_{i\ell }={Ũ}_{i}^{T}{Ṽ}_{\ell }+{\tilde{\epsilon }}_{i\ell },$$

where $\tilde{G}=CG,$ Ũ=CU, Ṽ=V, and where ${\tilde{\epsilon }}_{\ell }$ are statistically independent Gaussian vectors of mean zero and covariance matrix equal to identity. The matrix Ũ and Ṽ were obtained by applying a singular value decomposition of rank *K* to the transformed data matrix, *CG*. Then, *U* and *V* were obtained by applying a singular value decomposition of rank *K* to $C^{-1}ŨṼ$. To avoid multiple solutions, the orthogonality condition *VV^T^* = *I_K_*, where *I_K_* is the identity matrix in *K* dimensions, was imposed to *V* (Figure 1). The time needed to compute spFA is the same order as the time needed to compute *K* scores and loadings for a standard PCA (Patterson et al., 2006). For an example of implementation, see our R code^1^.

### Sparse factor analysis

Sparse Factor Analysis (SFA) was introduced by Engelhardt and Stephens (2010) as an alternative to admixture-based models, and this method can recapitulate the results of PCA when population structure is influenced by IBD patterns. To give a description of SFA, we considered a regression model of the following form

(4) $$G_{i\ell }=U_{i}^{T}V_{\ell }+\epsilon_{i\ell }$$

in which the residual errors are independent Gaussian random variables, ε*_i,ℓ_* ∼ *N*(0,1/*ψ_i_*), and where the prior distribution on the precision parameter, *ψ_i_*, is a Gamma distribution. In the SFA model, an automatic relevance determination prior is considered for the score vectors, $U_{ik}\sim N\left(0,\sigma_{ik}^{2}\right),$ where some $\sigma_{ik}^{2}$ are constrained to be equal to zero. We implemented SFA using the code distributed in Engelhardt and Stephens (2010), and we used 1,000 iterations. Eigenvectors in spFA and in SFA are also referred to as *factors* or *axes*.

### Simulated data

We generated simulated data for two diverging populations using coalescent models implemented in the computer program *ms* (Hudson, 2002). In these models, each population was simulated according to a linear stepping-stone model with 50 demes. To reproduce the simulation settings of Novembre and Stephens (2008), the effective migration rate between pairs of adjacent demes was set to the value 4*Nm* = 1. The divergence time τ between the two populations was varied within the range of values τ = (0,100) measured in coalescent units. We sampled 100 individuals, one from each deme both side of a (fictive) geographic barrier. For each simulation, we evaluated Wilks’ Λ, a statistic used in multivariate analysis of variance to test whether there are differences between the means of identified groups of individuals on the combination of genotypes (Mardia et al., 1979).

## Results

### Pure isolation-by-distance patterns

**Figure 2:**
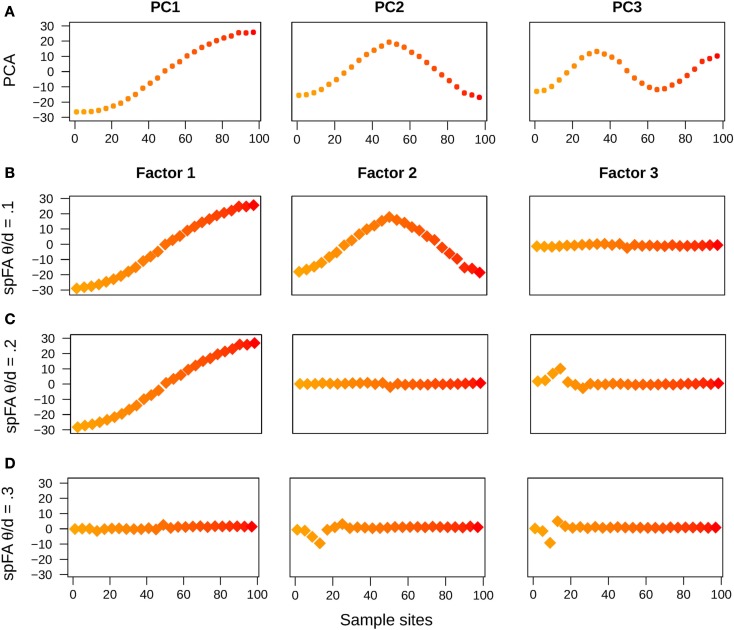
**PC and spFA factor maps for data simulated under an IBD model**. **(A)** PC maps, **(B)** spFA factor maps for $\theta ∕\bar{d}=0.1,$ **(C)** spFA factor maps for $\theta ∕\bar{d}=0.2,$ **(D)** spFA factor maps for $\theta ∕\bar{d}=0.3.$

In a first series of experiments, we used simulations of one-dimensional stepping-stone models reproducing the patterns of IBD described in Novembre and Stephens (2008). In those simulated data, the divergence time between the two populations was thus set to τ = 0, and the populations were connected by recurrent gene flow (4*Nm* = 1). As expected from theoretical results for PCA and for other ordination methods (Ahmed et al., 1974; Dray et al., 2006; Novembre and Stephens, 2008), the first PC maps displayed oscillating patterns. In addition, the frequency of oscillation increased as we examined axes of higher orders (Figure 2A). When we used sPCA, the first three positive components were almost identical to those obtained with PCA (not reported).

Running spFA with *K* = 3 and with 3 distinct values of the scale parameter ($\theta /\bar{d}=0.1$, 0.2, and 0.3) led to different interpretations of the genetic data (Figures 2B–D). Gradually varying *θ* allowed us to evaluate the scales at which the IBD effects were apparent, and also allowed us to remove those effects sequentially. For $\theta /\bar{d}=0.1$, the maps corresponding to factor 1 and 2 displayed sinusoidal curves similar to PC1 and PC2, whereas the map for factor 3 was flat as expected if the effect of IBD is removed (Figure 2B). For $\theta /\bar{d}=0.2$ the map corresponding to factor 1 remained similar to PC1, but the maps for factor 2 and factor 3 were flat (Figure 2C). For $\theta /\bar{d}=0.3$ the effects of isolation-by-distance were corrected in all axes (Figure 2D).

**Figure 3:**
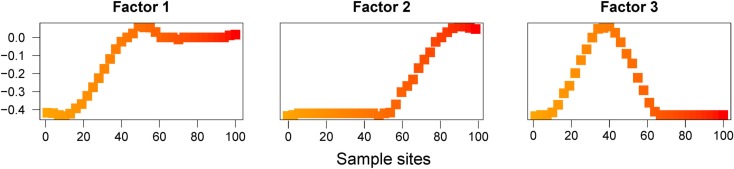
**SFA factor maps for data simulated under an IBD model**. Plots of the first three Factor maps for SFA.

When we ran SFA with *K* = 3 factors, the resulting maps also emphasized aspects of the data different from the ones described by PC maps and spatial factor maps (Figure 3). Maps for SFA are interpreted in terms of clusters, similar to those obtained in non-spatial Bayesian assignment programs like structure (Pritchard et al., 2000). Clusters created by clustering programs under IBD models are often reported as being undesirable (François and Durand, 2010; Meirmans, 2012).

### Two diverging populations with IBD patterns

In a second series of experiments, we used simulations of a two-population model, where each population consisted of a linear network of 50 demes. In these experiments, the two populations were separated by a geographic barrier to gene flow.

**Figure 4:**
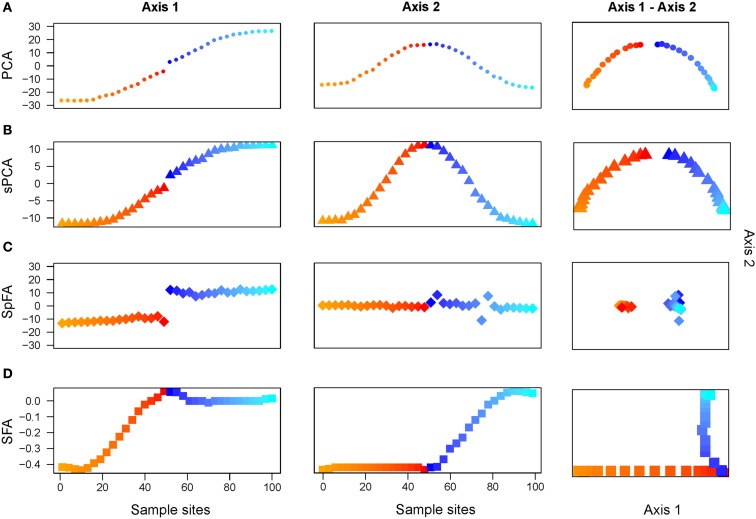
**Two discrete populations under equilibrium IBD**. Plots of the first 2 maps for **(A)** PCA, **(B)** sPCA, **(C)** spFA, **(D)** SFA.

First the divergence time was set to τ = 10 coalescent units. Using PCA, the first 2 components displayed oscillating patterns, similar to those obtained with τ = 0 (pure IBD simulations; Figure 4A). The PC1-PC2 plot exhibited a clear horseshoe pattern. Differentiation between the two populations was visible in the PC1 map, where a discontinuity was observed at the center of the habitat. This discontinuity corresponded to the localization of the geographic barrier. Results for the positive eigenvectors of sPCA strongly resembled those obtained for the first PCs (Figure 4B).

**Figure 5:**
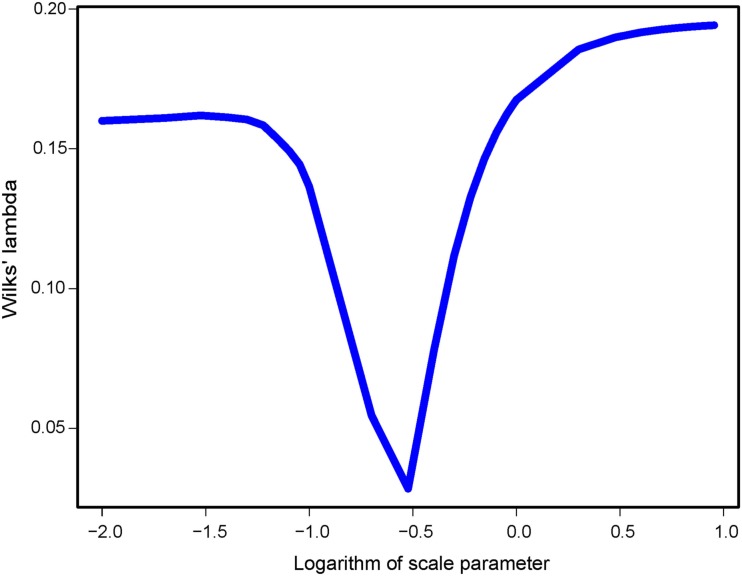
**Wilks’ Λ statistic as a function of the scale parameter $\theta ∕\bar{d}$ in spFA**.

Turning to spFA, we argued for a particular choice of $\theta ∕\bar{d}$ based on Wilks’ Λ statistic, a standard measure of separation of groups in discriminant analysis, and computed this statistic for $\theta ∕\bar{d}$ ranging between 0.01 and 10. As spatial factor analysis provided different interpretations of the data depending on the scale at which the data were analyzed, the choice of *θ* was crucial to the method. Figure 5 reports the value of Wilks’ Λ as a function of the logarithm of $\theta ∕\bar{d}.$ Values of $\theta ∕\bar{d}$ minimizing Wilks’ statistic and providing the best assignment of our data into clusters were about 0.32 (Figure 5). When spFA was applied with *K* = 2, the first factor map grouped demes at the left and the right of the geographic barrier in two main clusters, while simultaneously correcting for IBD patterns within the two clusters (Figure 4C). The spFA Axis1-Axis2 plot removed the horseshoe effect observed in PCA and sPCA plots. The resulting figure emphasized a discontinuous population structure consisting of two differentiated genetic clusters. Running SFA with *K* = 2 also led to a description of the data in two genetic clusters, located both sides of the geographic barrier, but the method failed to describe the two clusters as discontinuous entities (Figure 4D).

**Figure 6:**
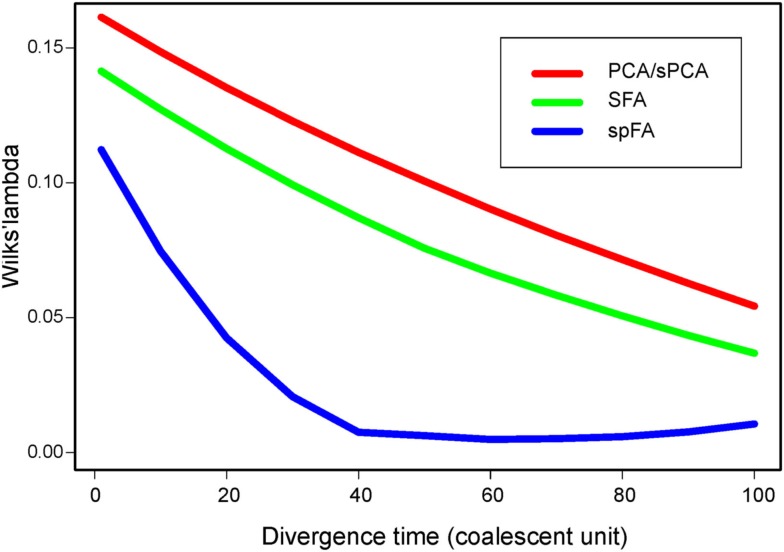
**Wilks’ Λ statistic as a function of the divergence time, τ, ranging between 1 and 100**.

Based on PC and factor plots, we next computed Wilks’ Λ statistic for all methods, and for divergence times τ ranging between 0 and 100 (Figure 6). Lower values of Λ generally indicated better discrimination of the 2 divergent populations in PC or factor plots. For all methods, the Λ statistic decreased as the divergence time between the 2 populations increased (McVean, 2009). In our spatially explicit framework, SFA (green curve) detected the existence of diverging populations earlier than PCA (red curve) and than sPCA (not shown, similar to PCA). SpFA was the most sensitive method, and provided an earlier detection of divergent clusters than SFA and PCA (blue curve).

### Human data analysis

**Figure 7:**
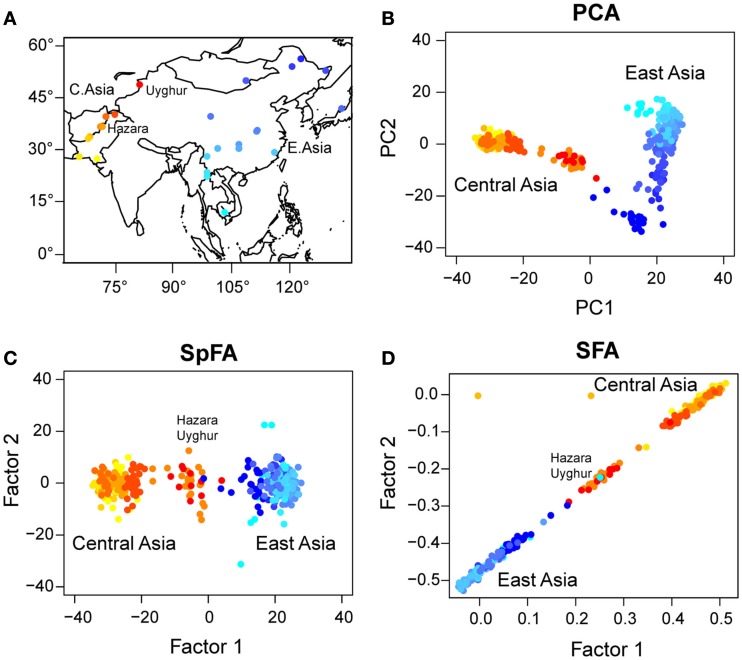
**(A)** Map of Asia with geographic locations of HGDP populations. PC and factor plots for **(B)** PCA, **(C)** spFA, **(D)** SFA.

Next we applied PCA, sPCA, spFA, and SFA to a worldwide sample of genomic DNA from 418 individuals in 27 Asian populations, from the Harvard Human Genome Diversity Project - Centre Etude Polymorphism Humain (Harvard HGDP-CEPH)^2^. In those data, each marker has been ascertained in samples of Mongolian ancestry (referenced population HGDP01224). We selected all samples from Central and East-Asia with the exception of Xibe, which originated in northeastern China, but migrated to northwestern China only recently (Powell et al., 2007) (Figure 7A). The data set used a panel of 10,664 SNPs^3^ (see Patterson et al., 2012).

**Figure 8:**
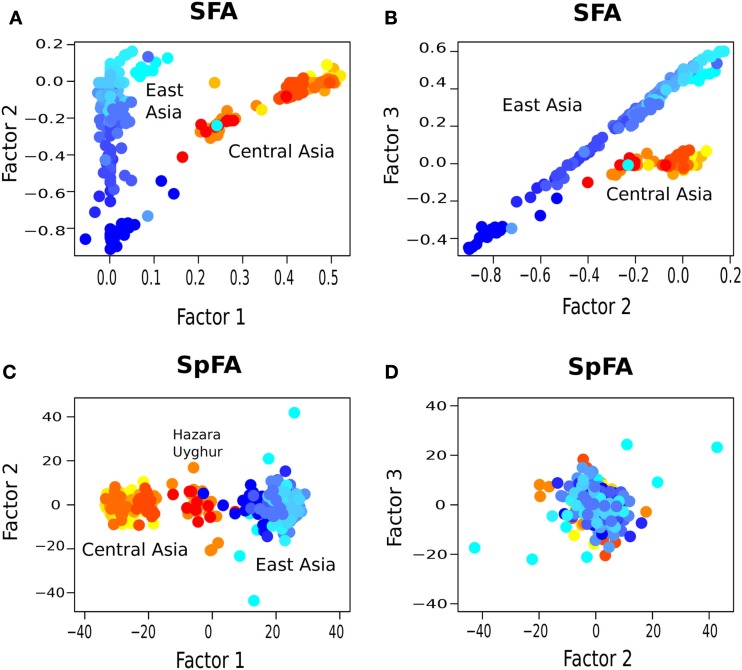
**Factor plots for (A,B) SFA and (C,D) spFA with *K* = 3 clusters**.

In our analysis, samples from Central Asia, west to the Tibetan plateau, were represented with red/orange colors, whereas populations from East-Asia were represented with blue colors (Figure 7A). For those samples, the PC plot exhibited a horseshoe pattern, which was a signature of the presence of IBD patterns in the data (Figure 7B). PCA led to a continuum of samples without observable genetic discontinuities. Running spFA with *K* = 2 and setting $\theta ∕\bar{d}=10^{-2}$ on the basis of Wilks’ statistic analysis, spFA corrected for the effects of IBD in axes 1 and 2 (Figure 7C). The spFA method provided evidence of a major discontinuity separating two clusters, one in Central Asia and one in East-Asia. In addition, Uyghur and Hazara population samples aligned with the two main clusters and were placed in an intermediate position, suggesting genetic admixture from ancestral Central Asian and East-Asian gene pools. Essentially the same patterns emerged when spFA was applied with *K* = 3 at the same scale (Figures 8C,D).

Using SFA with *K* = 2, factors 1 and 2 confirmed the main discontinuity, in a representation of clusters closer to Bayesian clustering methods than to PCA (Figure 7D). Uyghur and Hazara population samples were also placed between the main clusters. When we used SFA with *K* = 3, we obtained shapes without natural interpretations (Figures 8A,B). SFA detected additional discontinuities whereas the other methods suggested that continuous genetic variation in geographic space was predominant.

## Discussion

Principal component analysis and related methods used to describe genomic variation among large population samples are known to produce results that can be distorted by IBD, and that may thus be difficult to interpret. The horseshoe effect is one of the distortions observed in PC plots that arises when covariance between allele frequencies decays exponentially with geographic distance. In this case, there is an established mathematical correspondence between the eigenvectors of the covariance matrix and the columns of a discrete cosine-transform (Ahmed et al., 1974; Diaconis et al., 2008). In this study, we used this correspondence to propose a new approach based on spatial models for the covariance structure of residual errors in factor analysis. In spFA, IBD effects were modeled through the introduction of a covariance matrix that accounts for the geographic distance between individuals explicitly.

We compared spFA to PCA and to two recent methods that also attempt to correct for IBD effects: spatial Principal Component Analysis (sPCA, Jombart et al., 2008) and sparse factor analysis (SFA, Engelhardt and Stephens, 2010). When we applied PCA to simulated data from spatial coalescent models, PC maps displayed sinusoidal curves as observed in previous studies (Novembre and Stephens, 2008). We observed that sPCA, which includes several distance matrices within Moran eigenvector maps of genetic data, produced results similar to those of PCA, and did not correct for IBD effects. When we applied SFA to spatial coalescent simulations, the algorithm clustered individuals in several small groups depending on the number of latent factors used in the method. SFA factor maps actually displayed outcomes closer to discrete clusters than to continuous variation. After adjusting for the spatial scale in the covariance model, spFA was able to remove the oscillating shapes observed in the first PCs sequentially.

When PCA was applied to spatially explicit simulations of two diverging populations, PC maps failed to firmly identify genetic discontinuities between populations. Despite a relatively long period of isolation in simulations, the populations were not strongly separated in PC maps due to the horseshoe effect. Compared to PCA and sPCA, the spFA method had increased power to identify genetic discontinuities where they were masked by spurious autocorrelation effects. When we applied SFA, we found that, up to normalization of outputs, the results were similar to those generated by clustering algorithms like structure. For simulations of two diverging populations, SFA detected a main separation between two differentiated populations, but this approach did not correct for IBD effects within the main genetic clusters. Similarly to structure, the results of SFA were influenced by the presence of IBD patterns in the samples. We found that spFA alleviated this issue, and that it produced results more robust to the choice of the number of factors than SFA.

The methods used in this study provided quite distinct descriptions of the data when they were applied to human population samples from Central and East-Asia, and they underlined several aspects of the data. With PCA, a typical horseshoe pattern was observed, but no obvious genetic discontinuities were observed. In contrast, SFA provided evidence for two main clusters which were also confirmed by spFA. When we used SFA with *K* = 3, we obtained shapes without natural interpretations (Figure 8). SFA detected additional discontinuities whereas the other methods suggested that continuous genetic variation in geographic space was predominant. We observed that SFA behaves like clustering algorithms and did not correct for spurious clusters created by IBD patterns. This issue makes the SFA results difficult to interpret in terms of admixture and ancestral populations. The spFA method corrected for the horseshoe pattern observed in PC plots by removing autocorrelation effects from the second and third axes. The method suggested that Asian population structure is strongly influenced by IBD patterns. In the spFA plot, Hazara of Pakistan and Uygur of northwestern China grouped together, and were placed between Pakistani and East-Asian populations (Rosenberg et al., 2002). These results either support the presence of admixed genomes in Hazara and Uygur populations, or favor the hypothesis of a central Asian migration route of modern humans in East-Asia (Zhang et al., 2007). The public availability of data sets other than the HGDP will enable us to further assess the utility of the method for analyzing human genetic data.

A potential limitation of the spFA approach is it’s sensitivity to the choice of the scale parameter, *θ*. The *θ* parameter actually determines the scale of the spatial effects that could be removed by spFA. Note that spFA is essentially performing a standard principal component analysis when it is applied with small values of the scale parameter. In this study, we recommended exploring a grid of *θ* values so that IBD effects could be removed at distinct scales sequentially. The choice of the number of factors, *K*, in spFA is also tied to the particular value of *θ* implemented in the model. One way to determine *K* is by using Tracy-Widom tests on the matrix of genotypes, $\tilde{G}$ (Patterson et al., 2006). Gradually increasing the value of *θ* enabled a fine grain analysis of genetic discontinuities in human data, and allowed us to study IBD patterns within genetic clusters. The computational complexity of spFA increases linearly as a function of the number of markers. Since it is equivalent to the computation of a low rank approximation of the genotypic matrix (lower than a standard PCA, a few seconds on standard computer systems), applying spFA at multiple scales was not overly time-consuming.

## Conclusion

This study provided a comparison of existing methods that attempt to correct for IBD effects in population genetic analyses, and showed that each of studied approaches provided different insights on the data. Under equilibrium IBD, PCA was confounded by continuous variation and the main genetic discontinuities may be missed or misinterpreted. For the same data, SFA over-estimated the number of clusters in the genetic data, creating spurious clusters from continuous patterns. In the presence of IBD patterns, spatial factor analysis provided clearer interpretations of the data than PCA and SFA. In a spatially explicit framework, we found that spFA identified genetic discontinuities more efficiently than did PCA or SFA when these discontinuities are blurred by noise from IBD patterns in the genetic data.

## Conflict of Interest Statement

The authors declare that the research was conducted in the absence of any commercial or financial relationships that could be construed as a potential conflict of interest.

## Footnotes

^1^http://membres-timc.imag.fr/Olivier.Francois/spfa.R

^2^ftp://ftp.cephb.fr/hgdp_v3/

^3^ftp://ftp.cephb.fr/hgdp_supp10/
